# Avian haemosporidians in the cattle egret (*Bubulcus ibis*) from central-western and southern Africa: High diversity and prevalence

**DOI:** 10.1371/journal.pone.0212425

**Published:** 2019-02-22

**Authors:** Cynthia M. Villar Couto, Graeme S. Cumming, Gustavo A. Lacorte, Carlos Congrains, Rafael Izbicki, Erika Martins Braga, Cristiano D. Rocha, Emmanuel Moralez-Silva, Dominic A. W. Henry, Shiiwua A. Manu, Jacinta Abalaka, Aissa Regalla, Alfredo Simão da Silva, Moussa S. Diop, Silvia N. Del Lama

**Affiliations:** 1 Departamento de Genética e Evolução, Centro de Ciências Biológicas e da Saúde,Universidade Federal de São Carlos, São Carlos, SP, Brazil; 2 Percy FitzPatrick Institute, DST/NRF Centre of Excellence, University of Cape Town, Cape Town, South Africa; 3 Departamento de Ciências e Linguagens, Instituto Federal de Minas Gerais, Bambuí, MG, Brazil; 4 Departamento de Estatística, Centro de Ciências Exatas e Tecnológicas,Universidade Federal de São Carlos, Sao Carlos, SP, Brazil; 5 Departamento de Parasitologia, Instituto de Ciências Biológicas, Universidade Federal de Minas Gerais, Belo Horizonte, MG, Brazil; 6 A.P Leventis Ornithological Research Institute, Laminga, Jos, Nigeria; 7 Instituto da Biodiversidade e das Áreas Protegidas (IBAP), Bissau, Rep. da Guiné-Bissau; 8 AfriWet Consultants, Dakar, Thiaroye, Senegal; Justus-Liebeig University Giessen, GERMANY

## Abstract

We described the geographic distribution of 82 haemosporidian lineages (*Plasmodium*, *Haemoproteus*, and *Leucocytozoon*) in the cattle egret sampled in five countries in central-western and southern Africa. Seventy-three lineages have not previously been reported. We determined the prevalence of three haemosporidians in the samples. We investigated the influence of the internal environment of the host and environmental variables on the *Plasmodium* diversity and whether environmental variables may explain spatial variations in the prevalence of *Plasmodium*. We screened DNA from 509 blood samples from nestlings in 15 African colonies for infection by sequencing the cytochrome b gene of parasites. The molecular phylogenetic analysis was performed using Bayesian methods and including sequences from the MalAvi and GeneBank databases. We found 62 new *Plasmodium* lineages in a clade with MYCAME02, which is a lineage described in waterbirds and recently identified in birds of prey as *Plasmodium paranucleophilum*. Two *Haemoproteus* lineages identified in cattle egret formed a distinct group with *Haemoproteus catharti* and MYCAMH1 (*Haemoproteus* spp.). Seven *Leucocytozoon* lineages found in the cattle egret clustered with *Leucocytozoon californicus*. We found different *Plasmodium* diversities among the colonies sampled, demonstrating that the internal environment of the host is not the primary determinant of diversity. A linear mixed-effects multivariate model showed that precipitation was positively associated with *Plasmodium* diversity when controlling for the effects of temperature, colony composition (mixed and non-mixed species) and country. Moreover, a generalized mixed model showed that temperature was positively associated with the prevalence of *Plasmodium* when controlling for precipitation, elevation and country. We conclude that the cattle egret is a good model for future haemosporidian studies, as we found a significant number of new lineages in this host, which occupies regions with different climate characteristics where environmental variables exert an influence on the diversity and prevalence of *Plasmodium*.

## Introduction

Despite advances in studies on the genetic diversity, ecology and evolutionary biology of avian haemosporidians in recent years, factors driving the spatial variations of such parasites remain poorly understood. The spatial distribution of the diversity and prevalence of haemosporidians has been investigated using different hypotheses addressing a local or regional scale over a short or long period of time. Environmental hypotheses seek to explain the distribution of haemosporidian diversity and prevalence as a consequence of features of the external abiotic environment, such as climatic variables and altitude [1–3]. On the other hand, host hypotheses seek to explain the distribution and prevalence of haemosporidians based on host-related factors, such as composition of the host community, dispersal patterns [4, 5] and age of the host [6]. In fact, parasite distribution is simultaneously influenced by environmental, host and parasite-specific factors as well as vector abundance across multiple spatial and temporal scales [7, 8]. Detailed analyses of the biogeographic patterns of individual parasite assemblages on different spatial scales are important to understanding which environmental variables exert the greatest influence.

Several environmental factors have been identified as being directly involved in the incidence and transmission of avian blood parasites. Temperature was considered an important factor favouring the diversity and prevalence of *Plasmodium* spp. in a study involving 37 populations of the Iberian blackcap (*Sylvia atricapilla*) [9]. Sehgal et al. [10] showed that, among ecological variables, temperature was the strongest positive predictor of the prevalence of *Plasmodium* in the olive sunbird (*Cyanomitra olivacea*) sampled at 28 sites in central and western Africa. Rainfall has also been described as one of factors contributing to haemosporidian infection in wetlands in South Africa. Okanga et al. [11] demonstrated a significant correlation with rainfall two months prior to the sampling months and mosquito prevalence patterns across the landscape also demonstrated a close relationship to rainfall patterns. As Sehgal [3] points out, both temperature and rainfall exert a greater influence on vectors than bird hosts, since insects are ectothermic. Moreover, altitude has been associated with the occurrence of different genera and distribution patterns, with a predominance of *Leucocytozoon* at higher altitudes [2].

There are relatively few examples of single-species studies focusing on spatial patterns in the occurrence of haemosporidians [9, 12–15]. This approach enables comparing the effects of environmental factors on the prevalence and diversity of haemosporidians. However, this method is limited by the distribution of the host species and it is therefore necessary to choose a widespread cosmopolitan bird that occupies different landscapes. Following this logic, we undertook a spatially extensive analysis of haemosporidians in African populations of the cattle egret (*Bubulcus ibis*, Ardeidae). This gregarious egret normally breeds in colonies with other ardeid species near bodies of water [16], which favours the transmission of these parasites, since water availability is critical to the development of vector larvae [5]. Cattle egret nestlings are only partially covered with down upon hatching and chicks are not fully feathered until reaching 13 to 21 days of age [17], making them vulnerable to haemosporidian infection via mosquitoes and other biting flies that breed on or near water. The remarkably high diversity of avian malaria parasites and, consequently, the even higher number of potential parasite–host species interactions may modulate infection, especially the prepatent period and parasitemia dynamics [18]. Parasites can be detected in cattle egret nestlings aged two to four weeks, which likely reflects a very short prepatent period as a consequence of particular haemosporidian lineages infecting these birds. An experimental study has demonstrated that *P*. *ashfordi* and *P*. *relictum* lineages differ substantially in several life-history traits, especially the prepatent period and parasitemia dynamics [18]. Parasites can be detected in cattle egret nestlings aged two to four weeks, probably infected through transmission by vectors from adult to nestling (parent to offspring) or nestlings to nestlings in the nest or surrounding area in the colony.

In this study, we screened for three genera of haemosporidians (*Plasmodium*, *Haemoproteus* and *Leucocytozoon*) in cattle egret nestlings from 15 breeding colonies in central-western and southern Africa. Lineages of *Plasmodium*, *Haemoproteus* and *Leucocytozoon* were identified through a phylogenetic analysis that included previously described lineages. We determined the prevalence of three haemosporidians in the regions sampled. Although all three genera of parasites were detected, only *Plasmodium* was widely distributed, which enabled us to test the following hypotheses regarding its diversity and prevalence: H1 (host-diversity hypothesis)–the internal environment of the host is the primary determinant of *Plasmodium* diversity and, it is expected to find no variation in the composition of lineages found in cattle egret populations from different locations; H2 (environment-diversity hypothesis)–the external environment is the primary driving force of *Plasmodium* diversity in the cattle egret due to the likely impact of temperature, precipitation, beyond the effect of the colony composition (mixed and non-mixed species) on diversity. In this case, we expect *Plasmodium* diversity to be greater in regions with higher temperatures and greater precipitation as well as in mixed colonies, which are all factors that favour the transmission and interchange of parasites and, consequently, diversity; H3 (environment-prevalence hypothesis)–to test whether the variation in the prevalence of *Plasmodium* is explained by environmental effects, we suppose that the external environment influences the quantity of individuals that are infected and, in this case, we expect greater prevalence rates in regions with higher temperatures, lower elevation and greater precipitation, since these factors favour *Plasmodium* transmission.

## Material and methods

### Ethics statement

This study was conducted in strict accordance with African laws for research on wild birds in each country. All blood samples were collected with legal permission: Senegal (Ministere de LÉnvironnement et la Protection de la Nature, Direction des Eaux, Forets et Chasses, Numbers 01898, and 00064, Direction des Parcs Nationaux Number 00001274), Guinea-Bissau (Ministerio da Agricultura e Desenvolvimento Rural, 12/10/2011), Ghana (012174, 06/10/2011), and South Africa (Cape Nature AAA007 00010–0035, 07/11/2006). In Nigeria, it is not necessary to obtain a permit to collect and export blood samples from “least concern species”, as the cattle egret is classified. All methods related to capturing, handling, banding the birds and blood collection complied with internationally standardised sampling techniques [19].

### Study species and sampling

The subspecies *Bubulcus ibis ibis* originally occurred in central-eastern Africa [20], southern Europe, the Middle East, and parts of Asia [21–23]. It has historically dispersed with rapid growth and colonisation, expanding from its native range in Africa [22]. This subspecies currently occupies the entire African continent, with the exception of desert areas [24, 25].

Blood samples were collected from cattle egret nestlings (N = 509) at 15 breeding colonies in five countries in central-western and southern Africa: Senegal, Guinea-Bissau, Nigeria, Ghana and South Africa (S1 Table). Blood was collected (0.1 ml) from the right jugular or ulnar vein in the wing, using a syringe previously washed with anticoagulant (0.3% EDTA). Blood smears were taken from part of the samples collected in Senegal, Guinea-Bissau, Ghana and South Africa, totalling 224 individuals (81 from Senegal, 55 from Guinea-Bissau, 38 from Ghana, and 50 from South Africa). Four blood smears were prepared for each sample and were examined for 10–15 min at low magnification (×400). Two hundred fields were then studied at high magnification (×1000) using an Olympus Microscope CX31 (Olympus Corporation, Tokyo, Japan). Intensity of infection was estimated as a percentage by counting the number of parasites per 10,000 erythrocytes [26]. Parasites were identified by microscopy according to morphologic characteristics of blood stages [27]. We only examined blood smears from birds diagnosed as infected by haemosporidians using the molecular method.

### Molecular analyses

Total genomic DNA was extracted from blood samples using a phenol/chloroform/isoamyl alcohol protocol [28, 29]. DNA samples were screened for the presence of haemosporidians from three genera (*Plasmodium*, *Haemoproteus* and *Leucocytozoon*) with primers to amplify the conserved regions of the cytochrome b (cyt-b) gene [29] using a nested PCR protocol. After a screening PCR with HaemNF1 and HaemNR3 primers, the product was amplified in a second PCR reaction including HaemNR2 and HaemNF primers to identify *Plasmodium* and *Haemoproteus* and HaemL and HaemFL primers to identify *Leucocytozoon*. Two microlitres of the cyt-b amplicon obtained in the second-round PCR reaction were used as the template for the sequencing reaction. All negative reactions for haemosporidians were checked by another round of PCR. All PCR and sequencing reactions were performed in an Eppendorf Mastercycler Gradient thermal cycler (Eppendorf, Hamburg, Germany). The PCR products of DNA from all infected egrets were purified prior to sequencing by incubating 8.0 μL of the PCR product with 0.5 U of SAP enzyme (Shrimp Alkaline Phosphatase, USB) and 1 U of Exo I (Exonuclease I, USB) for 60 min at 37°C and 15 min at 80°C. The sequencing reaction was performed using 1 μL of Big-Dye (Applied Biosystems), 1 μL of purified DNA (50 ng/μL), 1 μL of primer (HaemF or HaemR2), 3 μL of buffer (200 mM Tris HCl, pH 9.0, 5 mM MgCl_2_) and 4 μL of sterile water. Excess dye-terminators were removed by ethanol precipitation. The product of the sequencing reaction was run in an ABI Prism 3730 or 3700 automatic sequencer (Life Technologies, Applied Biosystems, Hamburg, Germany).

### Phylogenetic analyses

The sequenced products of the cyt-b fragment (477 bp) from the haemosporidians were edited, aligned and analysed. All sequences were checked visually and trimmed using Codon Code Aligner software (CodonCode Corporation, Dedham, MA, USA). The sequences were aligned with ClustalX2 default parameters [30]. The substitution model was chosen using the Akaike information criterion [31] implemented in the MrModelTest 2.3 program [32]. The best-fit model was selected as general time-reversible + proportion of invariable sites + gamma distributed rate across sites (GTR + I + G) [33].

Lineages with 1 bp difference were considered evolutionary independent lineages. Prior to the phylogenetic reconstruction, we used the BLASTN tool to compare similarity among the *Plasmodium*, *Haemoproteus*, and *Leucocytozoon* cyt-b sequences obtained from the *B*. *ibis* samples and sequences of cyt-b lineages deposited in the MalAvi database [34] to determine whether there were previously described lineages (assigned or not to a morphospecies) with 100% similarity to those found in *B*. *ibis*.

*Plasmodium* and *Haemoproteus* cyt-b sequences were then separated from the *Leucocytozoon* sequences to perform two independent phylogenetic reconstructions among *B*. *ibis* lineages and morphospecies lineages described in MalAvi database [34]. The reason for partitioning the phylogenetic reconstruction into two independent analyses (*Plasmodium* + *Haemoproteus* and *Leucocytozoon*) was to enable better visualisation among the lineages from *B*. *ibis* described here and the haemosporidian morphospecies related to the development of avian malaria and haemosporidian-related parasites. In addition to the morphospecies described in MalAvi, we have only included non-described *Haemoproteus* lineages related to the new clade of haemosporidians described by Yabsley et al. [35]. Bayesian inference was performed using MrBayes 3.2.0 [36] to reconstruct the two phylogenetic hypotheses and was run for ten million generations, with trees sampled every 100 generations (the first 25,000 trees were discarded as ¨burn in¨). As suggested in the specialised literature, we used *Leucocytozoon toddi* as the outgroup [37].

Since nucleotide differences among most *Plasmodium* lineages found in *B*. *ibis* were very small, we considered that using a haplotype network method would be more effective than a phylogenetic tree-based approach to recover and depict relationships among *Plasmodium* lineages. We used the median-joining network approach to infer the haplotypes relationship using Network Software v.5.0.0.1[38].

### Statistical analyses

Lineages of haemosporidians are defined by different sequences of DNA and diversity can be evaluated based on nucleotide diversity, which is defined as the average number of nucleotide differences per site between two DNA sequences in all possible pairs in the sample population. Nucleotide diversity based on *Plasmodium* spp. lineages was determined for each colony population using the DNAsp program, version 5 [39]. An ANOVA model was used to test differentiation among the nucleotide diversity values, with variances given by the estimates provided by the DnaSP program, version 5 [39].

The prevalence of *Haemoproteus*, *Leucocytozoon* and *Plasmodium* was calculated for each cattle egret colony. Prevalence rates of *Plasmodium* determined in the colonies were compared using the chi-squared test.

Two environmental variables (average temperature and precipitation during the sampling month) were compiled using the WorldClim 1.4 at a 30-second resolution (http://www.worldclim.org) for geographic coordinates relative to the breeding colonies. To evaluate the joint effect of environmental variables on diversity, we employed a linear mixed-effects model using temperature, precipitation and type of colony (mixed species colonies versus single species colonies) as fixed effects and country as a random effect [40]

A generalized linear mixed-effects model with a binomial family and logit link was used to understand the relationship between prevalence and environmental variables [40]. We used temperature, precipitation, and elevation as fixed effects and country as a random effect. All analyses were performed using the R programme [41]. Plots were made using the tidyverse package (https://www.tidyverse.org). All the other analyses were performed using base R functions as well as the lme4 package [42].

## Results

To validate the identification of haemosporidians, we performed a morphological analysis of 224 blood smears in parallel with the positive molecular diagnosis using DNA. Sixty-four cases of *Plasmodium* infection were confirmed in the cattle egret smears (28 from Senegal, 16 from Guinea-Bissau, 11 from Ghana, and nine from South Africa). In this positive group, eight blood smears were found with trophozoites similar to *Plasmodium paranucleophilum*, but the confirmation of this species as involved in the infection was not possible due to the low level of parasitaemia detected in the nestlings. As expected, we found submicroscopic infection (samples that were positive by PCR, but negative on smears). With positive smears, parasitemia was low and it was therefore not possible to observe the different forms of the parasites for the confirmation of the species of *Plasmodium* involved in the infection. Infections by the genus *Leucocytozoon* were found mainly in Nigeria, but no smears were made in this location and these infections were only detected using the molecular approach.

### Diversity of lineages

**Fig 1:**
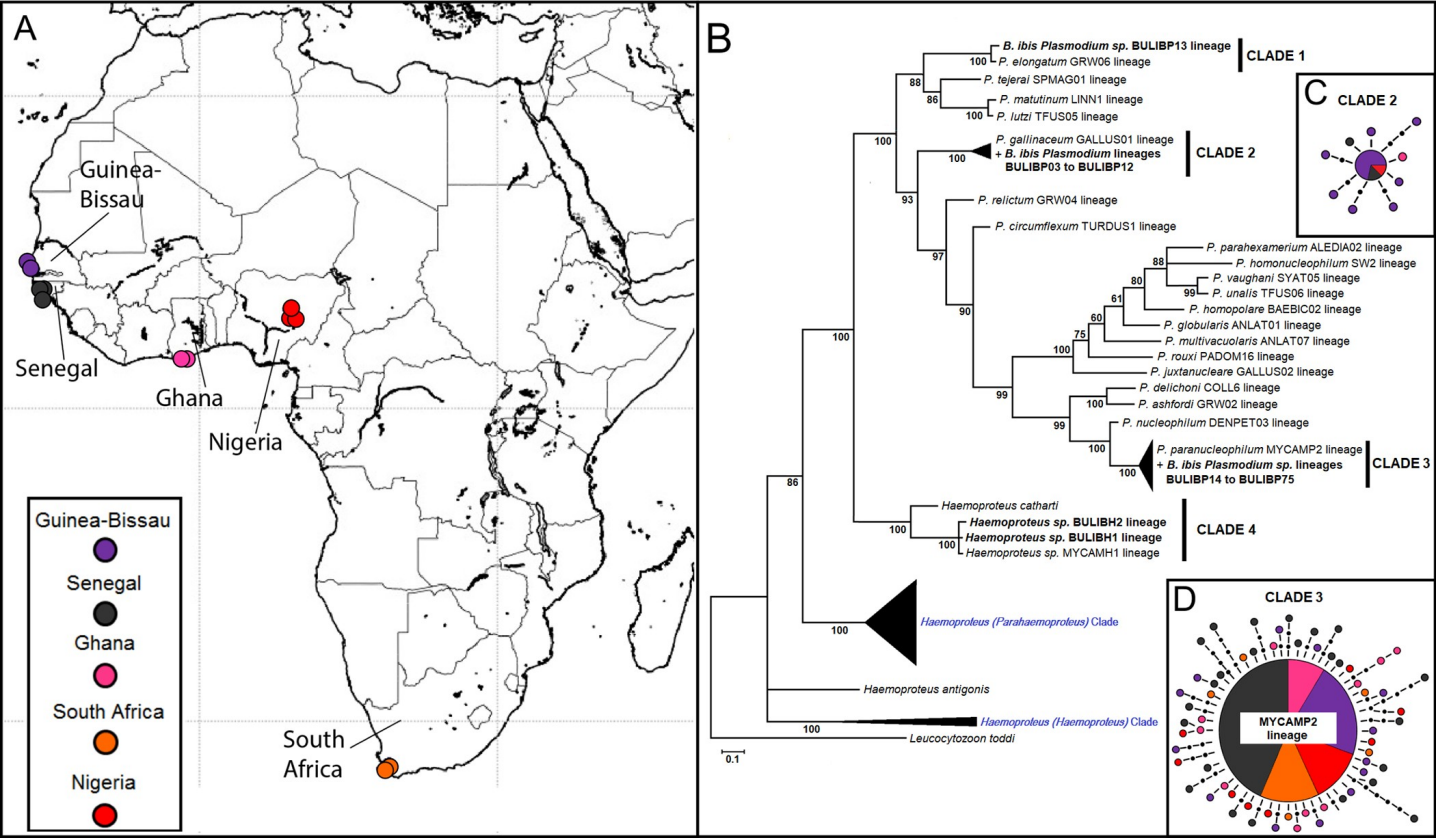
Location of sampling sites in Africa and phylogenetic identification of haemosporidian lineages. A) Map of Africa with sites where blood was sampled from cattle egret nestlings. B) Phylogenetic tree defined by Bayesian method showing lineages identified in this study and lineages from literature and database. C) Haplotype network corresponding to *Plasmodium* spp. lineages from BULIBP3 to BULIP12 from clade 2. D) Haplotype network showing genetic relationships among *Plasmodium* spp. lineages from BULIBP14 to BULIBP75 from clade 3.

Among the 509 blood samples tested (Fig 1A) using the molecular approach, 130 were positive for infection by *Plasmodium* spp., 35 were positive for infection by *Leucocytozoon* spp., and seven were positive for infection by *Haemoproteus* spp. Four samples of infected birds (two from Guinea-Bissau and two from Ghana) exhibited mixed infections with two different lineages of *Plasmodium*, which were detected by double peaks in the chromatograms. The first phylogenetic analysis involved cyt-b sequences from *Plasmodium* and *Haemoproteus* obtained from each infected cattle egret (N = 178), revealing two *Haemoproteus* and 73 *Plasmodium* lineages (Fig 1B, 1C and 1D).

The cyt-b phylogenetic tree representing the relationships among *Plasmodium* and *Haemoproteus* lineages had four highly supported clades (1, 2, 3 and 4) (Fig 1B). All *Plasmodium* clades (1, 2 and 3) were well-supported branches and were inserted in the major *Plasmodium* division. Clade 1 (Fig 1B) joined the BULIBP13 lineage identified in Ghana and Guinea-Bissau to the cosmopolitan *Plasmodium elongatum* lineage GRW06, which was previously found in the cattle egret in Spain [43] and the Great Blue heron (*Ardea herodias*) [44]. Clade 2 (Fig 1B and 1C) included the BULIBP3 to BULIP12 lineages, the most prevalent of which was the BULIBP5 lineage, from which nine lineages derive. Clade 2 joined the cattle egret lineages with the GALLUS01 *Plasmodium* lineage previously identified in *Gallus gallus* (Galliforme, Phasianidae) and other species of the Galliformes and Passeriformes orders [45–47]. Clade 3 (Fig 1B and 1D) is the largest and the most derived clade, with a central BULIBP15 lineage, from which 61 lineages derive (24 from Senegal, 14 from Guinea-Bissau, 10 from Ghana, eight from Nigeria, and five from South Africa). The BULIBP15 lineage corresponds to the MYCAME02 lineage described in the wood stork [48] and recently identified as *Plasmodium paranucleophilum* in birds of prey [49]. The BULIBH1 and BULIBH2 lineages belong to Clade 4, the branch topology of which in the tree (Fig 1B) represents a well-supported group of the *Haemoproteus* group, distinct from the *Parahaemoproteus* group. Clade 4 included the cattle egret lineages, the MYCAMH1 lineage described in the wood stork [48], and *Haemoproteus catharti*, which has recently been characterised in New World vultures [35]. The group of seven *Leucocytozoon* lineages were identified in a distinct phylogenetic tree (S1 Fig) using *Plasmodium juxtanucleare* as the outgroup. Seven *Leucocytozoon* lineages joined in a clade with *Leucocytozoon californicus* described in the American kestrel (*Falco sparverius sparverius*) using molecular and microscopic approaches [50]. Among the 82 lineages described, the BULIBH1, BULIBP5, BULIBP13, BULIBP15, BULIBP42, and BULIBL77 lineages are classified as generalists, since they have been found in other avian genera and families (S2 Table).

### Diversity by geographic location

**Fig 2:**
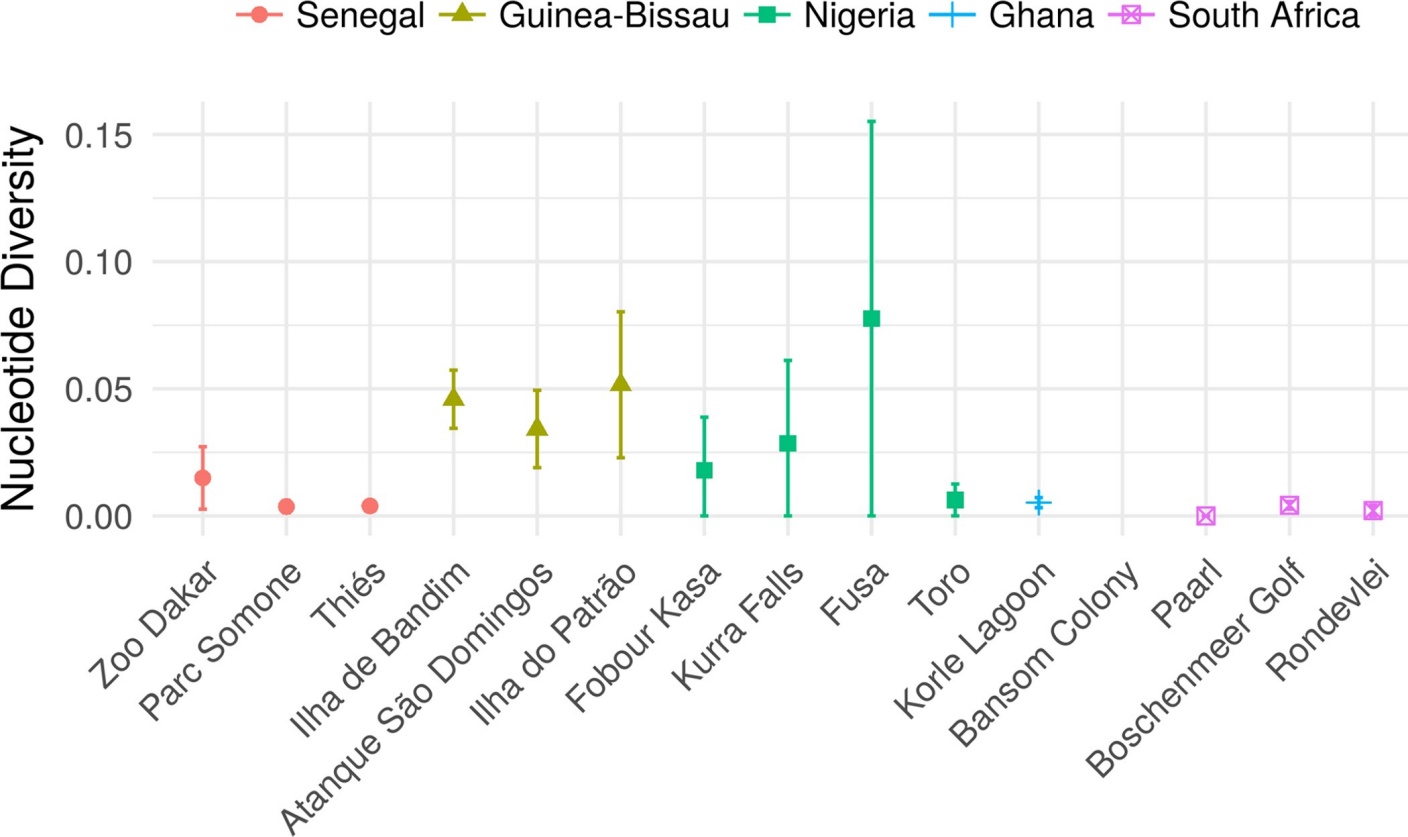
Spatial distribution of *Plasmodium* spp., nucleotide diversity, and relationships with environmental variables. Nucleotide diversity and variances of fifteen cattle egret colonies from five countries in central-western and southern regions of Africa.

Nucleotide diversity of *Plasmodium* lineages varied across colonies, and this variance was small in most of the sites sampled (Fig 2). Moreover, the statistical test demonstrated significantly different nucleotide diversities among locations (p-value < 0.001). The paired test showed that diversity levels are not associated with geographic location.

A linear mixed-effects multivariate model showed that only precipitation was positively associated with *Plasmodium* diversity when controlling for the effects of temperature, colony composition (mixed and non-mixed species) and country (Table 1). The p-value for testing whether the variance of the random effect factor (country) is zero was close to 1, which indicates that geographical location is not associated with diversity.

**Table 1:** Parameter estimates from a linear mixed-effects model for evaluating the effects of environmental variables. The effect of emperature, precipitation and colony composition (mixed-species or species-specific) were evaluated on *Plasmodium* diversity (nucleotide diversity).

Variable	Estimate	SE^1^	DF^2^	t value	P > t
Intercept	-0.01287	0.03469	10	-0.37107	0.71833
Colony composition^3^	-0.00548	0.01058	10	-0.51797	0.61575
Temperature	0.00027	0.00154	10	0.17383	0.86547
Precipitation	0.00003	0.00001	10	2.90967	0.01557

^1^SE: Standard error.

^2^DF: Degrees of freedom.

^3^Colony composition: mixed colonies (Zoo Dakar, Somone, Ilha de Bandim, Atanque, Ilha do Patrão, Korle Lagoon, Boschnemeer Golf, and Rondeley) and non-mixed colonies (Thiés, Fobour Kasa, Falls, Fusa, Toro, Banson, and Paarl).

### Prevalence by geographic location

**Fig 3:**
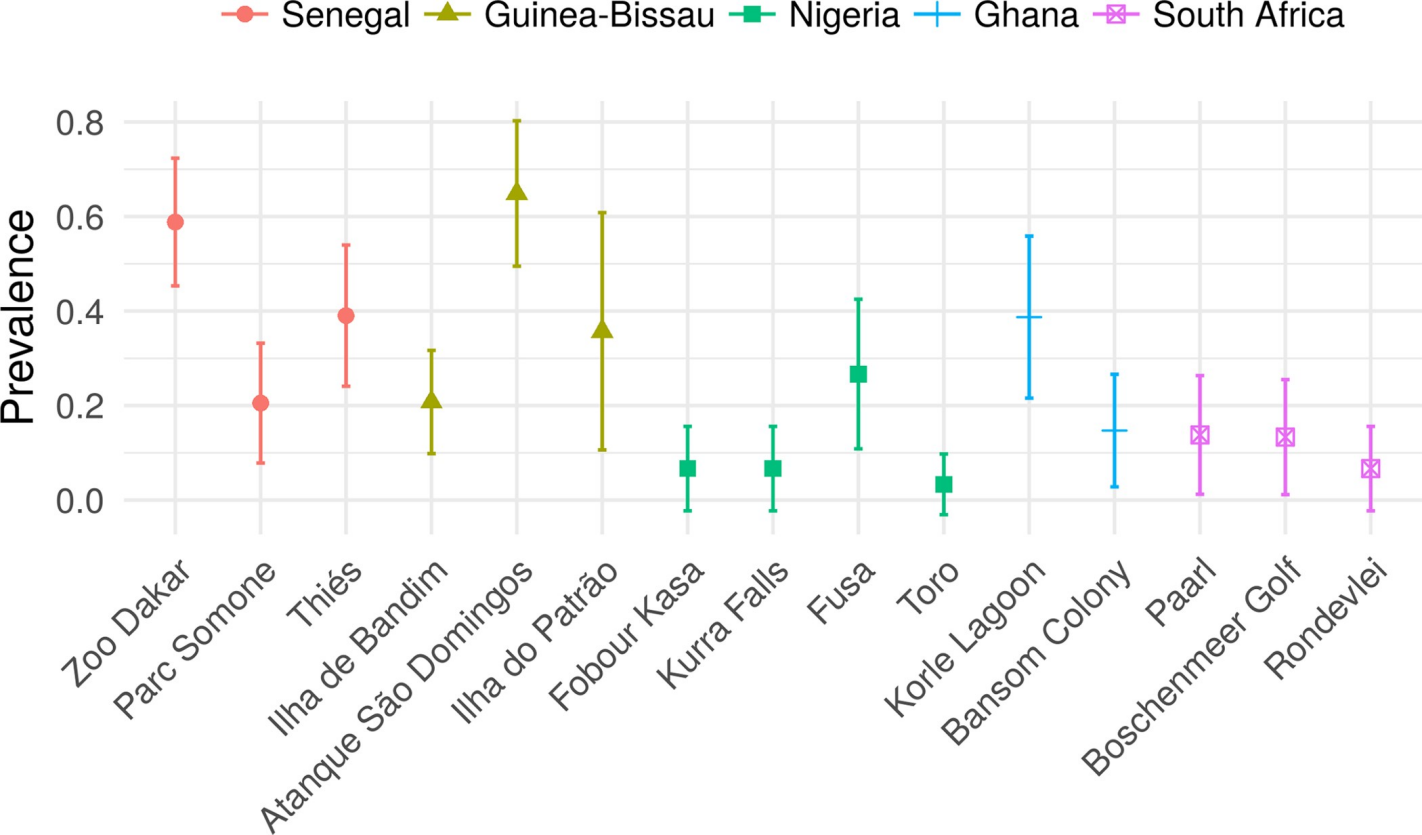
Spatial distribution of prevalence and relationships with environmental variables. Prevalence of *Plasmodium* with approximate 95% confidence intervals (computed using Gaussian approximation) in cattle egret colonies from five countries in central-western and southern Africa.

The geographic distribution of the prevalence of haemosporidians differed among three haemoparasite genera and colonies (p < 0.001) (Table 2 and Fig 3). *Plasmodium* dominated infections in moist/humid regions and lowlands areas. The presence of *Haemoproteus* was low in all regions sampled and *Leucocytozoon* was found mainly, but not exclusively at higher altitudes (p < 0.001) (Table 2). The statistical pairwise test showed that some of the colonies have significantly different *Plasmodium* prevalence values, but the levels were not clearly associated by geographic location.

**Table 2:** Prevalence of three genera of haemosporidians in fifteen cattle egret colonies in central-western and southern Africa and environmental variables at these sites. Environmental variables were extracted from WorldClim 1.4 database (http://www.worldclim.org): average temperature during sampling month, precipitation during sampling month and altitude of sampling sites.

Country	Colony (Population)	N birds	P-Infected birds	*Plasmodium* Prevalence	H-Infected birds	*Haemoproteus* Prevalence	L- Infected birds	*Leucocytozoon* Prevalence	Average Temperature (°C)	Precipitation (d^2^)	Altitude (mt)
Senegal	Zoo Dakar	51	30	0.588	0	0.000	0	0.000	27.65	124.00	9.0
Senegal	Somone	39	8	0.205	0	0.000	0	0.000	27.41	145.00	5.0
Senegal	Thiés	41	16	0.390	0	0.000	0	0.000	27.77	145.00	70.0
Guinea-Bissau	Bandim	53	11	0.208	2	0.038	1	0.019	26.37	358.00	22.0
Guinea-Bissau	Atanque	37	24	0.649	0	0.000	0	0.000	26.38	324.00	11.0
Guinea-Bissau	Ilha do Patrão	14	5	0.357	1	0.071	0	0.000	26.40	429.00	0.0
Ghana	K. Lagoon	31	12	0.387	0	0.000	0	0.000	27.13	80.00	23.0
Ghana	Bansom	34	5	0.147	0	0.000	0	0.000	25.56	211.00	103.0
Nigeria	Fobour Kasa	30	2	0.067	0	0.000	5	0.167	20.63	247.00	1,228.0
Nigeria	Kurra Falls	30	2	0.067	0	0.000	4	0.133	20.05	245.00	1,299.0
Nigeria	Fusa	30	8	0.267	0	0.000	3	0.100	20.00	252.00	1,303.0
Nigeria	Toro	30	1	0.033	1	0.033	11	0.367	22.05	244.00	937.0
South Africa	Paarl	29	4	0.138	1	0.034	5	0.172	17.72	39.00	97.0
South Africa	Boschenmeer	30	4	0.133	0	0.000	0	0.000	17.27	45.00	128.0
South Africa	Rondevlei	30	2	0.067	1	0.033	0	0.000	18.70	18.00	5.0

The generalized mixed model (Table 3) showed that the odds ratio between prevalence and temperature is 0.1681 (p < 0.001), meaning that the odds that a colony is infected increased by 18.31% with each degree centigrade increase in temperature. According to this multivariate model, altitude and precipitation were not associated with prevalence. Moreover, the p-value for testing whether the variance of the random effect factor (country) is zero was 0.667, which indicates that geographical location is not associated with prevalence.

**Table 3:** Parameter estimates from a generalized linear mixed-effects model evaluating the influence of environmental variables. The effect of temperature, altitude and precipitation on *Plasmodium* prevalence was evaluated.

Variable	Estimate	SE^1^	z	P > z
Intercept	-4.35001	1.14892	-3.78618	0.00015
Temperature	0.16812	0.04618	3.64065	0.00027
Altitude	-0.00055	0.00036	-1.51060	0.13089
Precipitation	-0.06203	0.03469	-1.78811	0.07376

^1^SE: Standard error.

## Discussion

We described 82 haemosporidian lineages using the mitochondrial cyt-b gene: 73 lineages of *Plasmodium*, seven of *Leucocytozoon*, and two of *Haemoproteus*. These data surpass the largest number of lineages previously described in the MalAvi database for a single species (66 lineages in *Parus major* from the order Passeriformes). The present results mainly contribute to the *Plasmodium* repertoire (69 new lineages), with lower proportions of *Haemoproteus* (one lineage) and *Leucocytozoon* (six lineages). The geographic distribution of the three genera is in agreement with the pattern described for African regions [51]. The genus *Leucocytozoon* was found almost exclusively at Nigerian sites, which are at higher altitudes: 23 infected birds were from this area among the 29 infected by this parasite. This pattern is in agreement with that described for the great tit (*Parus major*) in a study conducted at three altitudes in Switzerland, which demonstrated the predominance of *Plasmodium* at low altitudes and *Leucocytozoon* at high altitudes. Illera et al. [7] describe similar findings with 68 bird species in Iberian temperate mountains.

Studies on haemosporidians involving a single host bird species have reported a lower number of lineages in comparison to those found in the cattle egret. A study on the house wren (*Troglodytes aedon*) from the Andes and bordering lowlands revealed 23 distinct lineages [52], while twenty new lineages of haemosporidians were described in a raven (*Corvus corax*) population [15]. The order Ciconiiformes, which includes the cattle egret, had previously been described as having only 33 avian malaria lineages [34].

It is interesting to note that the new cattle egret haemosporidian lineages described here were united in clades of the phylogenetic tree with lineages that were recently characterised morphologically in different families of birds. In monophyletic clade 3 (Fig 1B), 62 cattle egret lineages were united with the wood stork lineage MYCAME02 [48] as well as *Plasmodium paranucleophilum* isolated and morphologically characterised in six species of birds of prey from the order Accipitriformes, Falconiformes and Strigiformes in Brazil [49]. The molecular identification performed in the present study is the first report of *P*. *paranucleophilum* in Africa, as this parasite has previously been reported only in South America [49]. The composition of clade 3 indicates that the *P*. *paranucleophilum* lineage similar to MYCAME02 has low specificity toward bird hosts and infects hosts from four orders of birds. Clade 4 clustered the two *Haemoproteus* lineages BULIBH1 and BULIBH2 with the MYCAMH1 lineage described in the wood stork and morphologically identified as *Haemoproteus catharti*, which infects turkey vultures [35]. Clade 4 is clearly separated from all other *Haemoproteus* species and had a position closer to *Plasmodium* species. The *Leucocytozoon* phylogenetic tree showed that cattle egret lineages (BULIBL76 to BULIBL82) joined with the *L*. *californicus* lineage in a cluster that is more closely related to leucocytozoid lineages found in owls (families Tytonidae and Strigidae) and passerines (families Fringillidae and Emberizidae) [50].

Among the 82 lineages identified from three genera of haemosporidians, only six lineages had the pattern of a generalist parasite (S2 Table). Based on Moens and Pérez-Tris [4], this low number of generalists found in the cattle egret would be expected, since the host species is abundant and specialist parasites have an advantage over generalist parasites because specialisation favours host monopolisation. The BULIBP5, BULIBP13, BULIBP15, BULIBP42 *Plasmodium* lineages occurred in the orders Accipitriformes, Ciconiiformes, Falconiformes and Strigiformes; BULIBH1, which is a *Haemoproteus* lineage, was found in the Ciconiidae and Cathartidae families, and BULIBL77, a *Leucocytozoon* lineage, was identified with the CIAE02 lineage, previously found in several bird orders (S2 Table) as well as the little bittern (*Ixobrychus minutes*, Ardeidae) [34].

Cattle egret has a wide distribution and underwent recent expansion on the African continent [53]. The haplotype network showed the most common haplotype in the central position, associated with few mutational steps to haplotypes with small frequencies, which may indicate a population expansion of *Plasmodium* along with the host species. There was no phylogenetic structuring of lineages by geographic region (Fig 1B, 1C and 1D). This is a pattern that is in line with connectivity among regions due to host movements, which leads to the homogenisation of parasites among regions. Most populations of the cattle egret are partially migratory, making long-distance dispersive movements to seek food resources and as a response to seasonal rainfall [54]. Hosts with wide distribution that are also migratory birds can encounter more parasites and often harbour a greater diversity of parasites in comparison to hosts with restricted distribution [55]. Thus, these migratory birds can spread pathogens, which can infect both migratory and resident species (S2 Table).

*Plasmodium* diversity differed among the sampling sites, which is not in agreement with the host hypotheses (H1 rejected) of spatial variation in parasite diversity and lends support to the environment hypothesis (H2 accepted). The multivariate model revealed that the composition of colony (mixed species) has no effect on diversity of *Plasmodium* lineages [56]. On other hand, the multivariate model (including temperature, precipitation and colony composition) showed that precipitation exerts a significant and positive association to *Plasmodium* diversity found among countries. The cattle egret chooses breeding and roosting sites near lakes and rivers [20], which offer standing water for the reproduction of *Culex* mosquitoes (*Plasmodium* vectors). Rainfall favours the multiplication of vectors in these water bodies, consequently contributing to transmission among birds and the increase in the diversity of *Plasmodium* lineages in the cattle egret population.

High *Plasmodium* prevalence rates (0.033–0.649) were found in the samples evaluated and demonstrate that cattle egrets may be infected early in life (two to four weeks), as Villar et al. [48] reports for the wood stork. The BULIBP15 lineage is the most prevalent *Plasmodium* lineage and was found in every colony sampled, except in the Boschenmeer Golf colony in South Africa. The prevalence of *Plasmodium* differed among sampling sites (H3 supported). The multivariate analysis showed that only temperature (among altitude, temperature and precipitation) is associated to *Plasmodium* prevalence when the other covariates are taken into account. The association between temperature and prevalence was expected. Temperature was reported to be the most important predictor of *Plasmodium* prevalence in the olive sunbird (*Cyanomitra olivacea*), which occupies several habitats in tropical Africa [10]. The lower prevalence rates found in cattle egret colonies located in South Africa is in agreement with what is expected for a temperate climate, where more severe winters and lower annual temperatures are common. Temperature fluctuation during the day has been described as an important determinant in the transmission of malaria [57]: warmer temperatures reduce the vector capacity of mosquitos [58], while lower temperatures limit the sporogonic development of *P*. *relictum* in the vector *Culex quinquefasciatus* [59]. Thus, *Plasmodium* spp. transmission is reduced at temperatures less than 13°C and more than 30°. As the daily fluctuations of temperature in most of the sites were within this range during sampling, this effect does not explain the results obtained. Cumming et al. [60] found the same effect of temperature on prevalence studying the repertoire of haemosporidians in eight species of duck (Anatidae) from four genera sampled from different areas of southern Africa. The authors found a lower prevalence rate of *Plasmodium* in the southernmost portion of the African continent (South Africa, S34o01’) and a higher prevalence rate in the northern portion of the region (Zimbabwe, S17o56’). Using samples from 12 bird species on an oceanic island (Tenerife, Canary Islands), Padilla et al. [1] found highest prevalence of *Plasmodium* in the lowest and warmest habitats. This finding is in agreement with the results of the present study, in which lower prevalence rates of *Plasmodium* were found in colonies located more inland and at higher altitudes in Ghana and Nigeria (Banson, Fobour Kasa, Kurra Falls, and Toro) as well as colonies located in the temperate zone (Bonshnemeer Golf, Paarl, and Rondeley in South Africa).

## Conclusions

We screened cattle egret populations located in the central-western and southern regions of Africa to detect the presence/absence of haemosporidian infection and found 82 lineages of three genera: *Plasmodium*, *Haemoproteus* and *Leucocytozoon*. The data on *Plasmodium* diversity demonstrated that the distribution of this variable was influenced by precipitation. The prevalence of genera differed among the fifteen locations where the hosts were sampled. Moreover, the prevalence of *Plasmodium* proved to be greatly influenced by temperature. The present findings demonstrate the importance of single-host studies for understanding the spatial variation of haemosporidians and validate the use of sedentary nestlings to determine the parasite assemblages at each location. The cattle egret proved to be a good model for the investigation of distribution and prevalence of haemosporidians, since it is a host with widespread occurrence, which enabled testing the influence of environmental variables.

## Supporting information

S1 Fig Bayesian phylogenetic tree to identify lineages of *Leucocytozoon*. All morphospecies used to identify these genera were downloaded from MalAvi (Bensch et al., 2009) and GenBank databases.
(DOCX)

S1 Table Geographic coordinates of African sites where blood samples were collected from cattle egret nestlings. Blood was collected from nestlings in breeding colonies. N is total number of birds sampled per colony.
(DOCX)

S2 Table Bird species that showed 100% similarity with new lineages described in cattle egret. BLASTN tool was used to compare the similarity among the *Plasmodium*, *Haemoproteus*, and *Leucocytozoon* cyt-b sequences obtained from the *B*. *ibis* samples and sequences of cyt-b lineages deposited in the MalAvi database. Lineages were classified as generalists when infecting avian species of different genera and families from *Bubulcus ibis*.
(DOCX)
