# Supplementary material for: Avian haemosporidians in the cattle egret (Bubulcus ibis) from central-western and southern Africa: High diversity and prevalence
Source: PLoS One. 2019 Feb 22;14(2):e0212425. doi: 10.1371/journal.pone.0212425 (PMC6386389; doi:10.1371/journal.pone.0212425)
Supplement: S1 Fig — All morphospecies used to identify these genera were downloaded from MalAvi (Bensch et al., 2009) and GenBank databases. (DOCX) [file pone.0212425.s001.docx]

**Supporting Information**


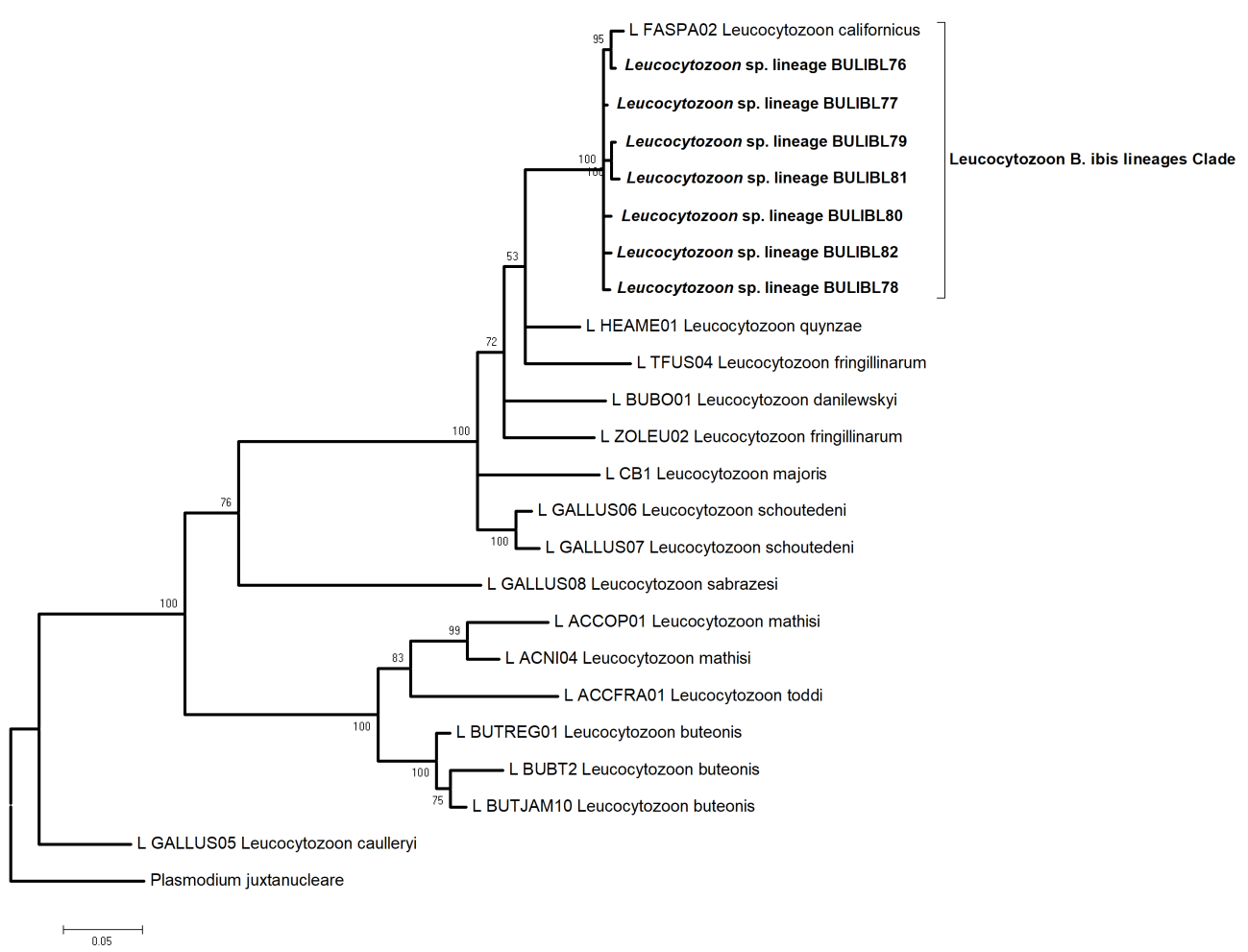


**S1 Fig. Bayesian phylogenetic tree to identify lineages of *Leucocytozoon*. A**ll morphospecies used to identify these genera were downloaded from MalAvi (Bensch et al., 2009 and GenBank databases.
