# Supplementary material for: Avian haemosporidians in the cattle egret (Bubulcus ibis) from central-western and southern Africa: High diversity and prevalence
Source: PLoS One. 2019 Feb 22;14(2):e0212425. doi: 10.1371/journal.pone.0212425 (PMC6386389; doi:10.1371/journal.pone.0212425)
Supplement: S1 Table — Blood was collected from nestlings in breeding colonies. N is total number of birds sampled per colony. (DOCX) [file pone.0212425.s002.docx]

**Supporting Information**

**S1 Table. Geographic coordinates of African sites where blood samples were collected from cattle egret nestlings**. Blood was collected from nestlings in breeding colonies, N is total birds sampled by colony.

| Country | Population | N | Geographic Coordinates | |
| --- | --- | --- | --- | --- |
| Senegal | Zoo Dakar | 51 | N1443.653’ | O1725.895’ |
|  | Somone | 39 | N1429.861’ | O1704.931’ |
|  | Thiés | 41 | N1447.789 | O1655.883’ |
| Guinea-Bissau | Ilha de Bandim | 53 | N1207.182’ | O0540.214’ |
|  | Atanque São Domingos | 37 | N1225.764’ | O1605.433’ |
|  | Ilha do Patrão | 14 | N1115.827’ | O1546.480 |
| Nigeria | Fobour Fasa | 30 | N0986.434’ | E0904.582’ |
|  | Kurra Falls | 30 | N0943.153’ | E0900.063’ |
|  | Fusa | 30 | N0987.991’ | E08 97.097’ |
|  | Toro | 30 | N1003.472 | E00903.864’ |
| Ghana | Korle Lagoon | 31 | N0506.309’ | O00123.359’ |
|  | Bansom Colony | 34 | N0506.475’ | O01854.810’ |
| South Africa | Paarl | 29 | S3341.051’ | E1859.031’ |
|  | Boschenmeer Golf | 30 | S3345.000’ | E18 59.000’ |
|  | Rondevlei | 30 | S3403.759’ | E18 29.725’ |
| Total | | 509 |  |  |
