# Supplementary material for: Avian haemosporidians in the cattle egret (Bubulcus ibis) from central-western and southern Africa: High diversity and prevalence
Source: PLoS One. 2019 Feb 22;14(2):e0212425. doi: 10.1371/journal.pone.0212425 (PMC6386389; doi:10.1371/journal.pone.0212425)
Supplement: S2 Table — BLASTN tool was used to compare the similarity among the Plasmodium, Haemoproteus, and Leucocytozoon cyt-b sequences obtained from the B. ibis samples and sequences of cyt-b lineages deposited in the MalAvi database. Lineages were classified as generalists when infecting avian species of different genera and families from Bubulcus ibis. (DOCX) [file pone.0212425.s003.docx]

**Supporting Information**

**S2 Table Bird species that showed 100% similarity with new lineages described in cattle egret.** BLASTN tool was used to compare the similarity among the *Plasmodium*, *Haemoproteus,* and *Leucocytozoon* cyt-b sequences obtained from the *B. ibis* samples and sequences of cyt-b lineages deposited in the MalAvi database. Lineages were classified as generalists when infecting avian species of different genera and families than *Bubulcus ibis*.

| **Generalist parasite lineage**  **this study** | **MalAvi**  **lineage name** | **Host** | **Host-status** | **G Geographic location** |
| --- | --- | --- | --- | --- |
| BULIBH1 (*Haemoproteus*) | MYCAME08 | *Mycteria americana* | Migratory | Brazil |
|  |  | *Mycteria americana* | Migratory | United States |
| BULIBP5 (*Plasmodium*) | GALLUS01 | *Gallus gallus* | *-* | Vietnam |
|  |  | *Gallus gallus* | Resident | Thailand |
|  |  | *Quiscalus quiscula* | *-* | United States |
|  |  | *Myiarchus swainsoni* | *-* | Brazil |
|  |  | *Myiarchus ferox* | *-* | Brazil |
|  |  | *Myiobius barbatu* | *-* | Brazil |
|  |  | *Corvus macrorhynchos* | *-* | Japan |
|  |  | *Passer montanus* | *-* | Japan |
| BULIBP13 (*Plasmodium*) | MILANS05 | *Milvus migrans* | *-* | Spain |
|  |  | *Philomachus pugnax* | *-* | Mali |
|  |  | *Philomachus pugnax* | *-* | Netherlands |
| BULIBP15 (*Plasmodium*) | MYCAME02 | *Mycteria americana* | Migratory | Brazil |
|  |  | *Cygnus atratus* | Resident | Brazil |
|  |  | *Phoenicopterus chilensis* | Resident | Brazil |
|  |  | *Micrastur semitorquatus* | Resident | Brazil |
|  |  | *Pseudoscops clamator* | Resident | Brazil |
|  |  | *Micrastur semitorquatus* | Resident | Brazil |
|  |  | *Pulsatrix koeniswaldiana* | Resident | Brazil |
|  |  | *Buteo brachyurus* | Resident | Brazil |
|  |  | *Buteo magnirostris* | Resident | Brazil |
|  |  | *Caracara plancus* | Resident | Brazil |
|  |  | *Falco peregrinus* | Migratory | Brazil |
|  |  | *Myiodynastes maculatus* | Resident | Brazil |
|  |  | *Setophaga petechia* | Migratory | United States |
|  |  | *Anas discors* | Migratory | United States |
|  |  | *Anas discors* | Migratory | Canada |
|  | MYCAME03 | *Mycteria americana* | Migratory | Brazil |
| BULIBP42 (*Plasmodium*) | MYCAME07 | *Mycteria americana* | Migratory | Brazil |
| BULIBL77 (*Leucocytozoon*) | CIAE02 | *Accipiter virgatus* | *-* | Philippines |
|  |  | *Ninox scutulata* | Migratory | Japan |
|  |  | *Milvus migrans* | *-* | Spain |
|  |  | *Circus aeruginosus* | *-* | Germany |
|  |  | *Gyps fulvus* | *-* | Spain |
|  |  | *Aegypius monachus* | *-* | Spain |
|  |  | *Crex crex* | Migratory | Poland |
|  |  | *Crex crex* | Migratory | Russia |
|  |  | *Buteo buteo* | Migratory | Turkey |
|  |  | *Buteo rufinus* | Resident | Turkey |
|  |  | *Asio otus* | Resident | Turkey |
|  |  | *Ixobrychus minutus* | Migratory | Turkey |
|  |  | *Falco eleonorae* | Migratory | Spain |
|  |  | *Larus cachinnans* | Migratory | Poland |
|  |  | *Larus argentatus* | Migratory | Poland |
|  |  | *Larus mongolicus* | Migratory | Mongolia |
|  |  | *Dendrocopos minor* | Resident | Portugal |
